# Silencing of LINC00707 suppresses cell proliferation, migration, and invasion of osteosarcoma cells by modulating miR-338-3p/AHSA1 axis

**DOI:** 10.1515/biol-2021-0070

**Published:** 2021-07-16

**Authors:** Xiao-rong Zhang, Jian-li Shao, Heng Li, Liang Wang

**Affiliations:** Department of Orthopedic and Traumatology, The First Affiliated Hospital of Jinan University, 601 West Huangpu Avenue, Tianhe District, Guangzhou 510630, China; Department of Orthopedic, Center in Zhanjiang City People’s Hospital, Zhanjiang 524045, China; Department of Oncology, The First Affiliated Hospital of Jinan University, 601 West Huangpu Avenue, Tianhe District, Guangzhou 510630, China

**Keywords:** LINC00707, osteosarcoma, ceRNA, miR-338-3p, AHSA1

## Abstract

Osteosarcoma is the most common type of primary malignant tumor of the bone, with a high metastatic rate and poor prognosis. Therefore, it is important to further elucidate the molecular mechanisms involved in the development of osteosarcoma and explore new molecular therapeutic targets. Long intergenic nonprotein-coding RNA 707 (LINC00707) is an oncogenic gene in several cancers. In this study, we further clarified its role and regulatory mechanism in osteosarcoma. We found that LINC00707 levels are significantly higher in the osteosarcoma cell lines SW 1353, HOS, U-2 OS, MG-63, and Saos-2 compared to those in human fetal osteoblastic cell line hFOB1.19. LINC00707 silencing suppressed cell proliferation, migration, and invasion of MG-63 and Saos-2 cells. Moreover, LINC00707 can act as a competitive endogenous RNA of miR-338-3p, and miR-338-3p inhibitor and AHSA1 overexpression alleviated the effect of LINC00707 silencing. In conclusion, we demonstrated high expression of LINC00707 in osteosarcoma cell lines and that silencing LINC00707 suppresses cell proliferation, migration, and invasion by targeting the miR-338-3p/AHSA1 axis in MG-63 and Saos-2 cells. These findings suggest that LINC00707 may serve as a potential target for osteosarcoma treatment.

## 1 Introduction

Osteosarcoma (OS) is a common malignant tumor that originates in the bone and mainly occurs in children and adolescents younger than 20 years [1]. Similar to other solid tumors, osteosarcoma has a high degree of malignancy and a poor prognosis [1]. Although surgical techniques and chemotherapy have made significant progress in the past few decades, the survival rate of OS patients has not improved much, and the 5-year survival rate is less than 70% [2]. Osteosarcoma is a disease with a complex genetic background and, so far, the pathogenesis- and tumorigenesis-driving genes have not been clarified. Currently, the treatment methods mainly include surgery, chemotherapy, and adjuvant chemotherapy [1]. Nonetheless, 30–40% of patients with osteosarcoma are at risk of lung metastasis and recurrence, and their prognosis is poor, with a 5-year survival rate less than 30% [3]. Therefore, it is crucial to explore new biomarker molecules for osteosarcoma and determine new, targeted treatment methods to improve the efficacy of osteosarcoma treatment.

Long noncoding RNAs (lncRNAs) are a type of noncoding RNAs with a length of more than 200 nucleotides [4]. They were first regarded as transcriptional “noise”; however, recent studies have shown that several lncRNAs can regulate gene expression in various biological processes. Abnormal expression of lncRNAs has been identified in various cancer tissues or cells [4,5]. Abnormally expressed lncRNAs are involved in tumorigenesis, progression, metastasis, and chemotherapy drug resistance [4,5]. It has been reported that long intergenic nonprotein-coding RNA 707 (LINC00707) plays an oncogenic role in lung adenocarcinoma, hepatocellular carcinoma, colorectal cancer, and breast cancer [6,7,8,9]. However, its role in osteosarcoma is unclear. In the present study, we focused on further analyzing the role of LINC00707 and regulatory mechanisms in osteosarcoma to provide new references for the diagnosis and treatment of osteosarcoma.

Recent studies have shown that lncRNAs are involved in several important regulatory processes such as chromosome silencing, genomic imprinting, chromatin modification, transcriptional activation, transcriptional interference, and nuclear transport [10,11,12]. Besides these, lncRNAs also affect the expression of their target genes by regulating microRNAs (miRNAs) expression [13]. miRNAs are noncoding RNAs containing about 22 nucleotides, which play a key role in posttranscriptional regulation of gene expression through binding to the 3′ untranslated region (UTR) of target mRNAs [14,15]. lncRNAs can be used as competitive endogenous RNA (ceRNA) to sponge miRNAs and thus regulate target gene expression. This regulatory mechanism is known as the ceRNA mechanism [13]. It has been reported that LINC00707 can act as a sponge of miR-206 and miR-485-5p [6,8]. However, we are far from fully understanding the mechanism of LINC00707 in cancer cells. In this study, we further investigated whether LINC00707 can act as ceRNA in osteosarcoma.

## 2 Materials and methods

### 2.1 Cell lines

The conditionally immortalized human fetal osteoblastic cell line hFOB1.19 and human osteosarcoma cell lines (SW 1353, HOS, U-2 OS, MG-63, and Saos-2) were purchased from the Institute of Cell Bank/Institutes for Biological Sciences (Shanghai, China). Cells were cultured according to our previous study [16].

### 2.2 Small interfering RNA transfection and cell groups

Small interfering RNA (siRNA) targeting LINC00707 (siRNA1: 5′-GGCUUUCCAUGACCCAUAAUU-3′, siRNA2: 5′-GGAAGCCACU CCUGCAUUUUU-3′, siRNA3: 5′-GCAGGAACAUCACCAUCUUUU-3′), negative control siRNA (si-NC), miR-338-3p mimic, negative control miRNA (miR-NC), miR-338-3p inhibitor, and miR-NC inhibitor were synthesized using GenePharma Co., Ltd (Shanghai, China). Lipofectamine RNAiMAX Reagent (Invitrogen, Carlsbad, CA, USA) was used for cell transfection according to the manufacturer’s instructions. To analyze the effect of LINC00707 silencing, MG-63 or Saos-2 cells were transfected with siNC or siRNA1 and classified as the siNC group and the siRNA1 group. To analyze whether the miR-338-3p inhibitor weakens the effect of LINC00707 silencing, MG-63 or Saos-2 cells were transfected with siRNA1 plus miR-338-3p inhibitor group or siRNA1 plus miR-NC inhibitor and classified as the siRNA1 + miR-338-3p inhibitor group and the siRNA1 + miR-NC inhibitor group. After transfected at 24 h, LINC00707 expression was measured by quantitative reverse transcription PCR (qRT-PCR).

### 2.3 qRT-PCR

Total RNA isolation and qRT-PCR to detect LINC00707 and miR-338-3p expression as well as their primer sequences were performed as previously described [16]. To determine the LINC00707 and miR-338-3p levels, 1 μg total RNA was reverse transcribed using random primers and M-MLV reverse transcriptase (Promega, Madison, WI, USA). qPCR was performed using SYBR Green qPCR SuperMix (Invitrogen, Carlsbad, CA, USA) on ABI PRISM^®^ 7500 Sequence Detection System (Foster City, CA, USA). The PCR reaction conditions have been described previously [16]. Gene expression was measured in triplicate and quantified using the 2^−ΔΔCt^ method. GAPDH was used as an internal control. The primer sequences of LINC00707 were as follows: LINC00707 forward 5′-CCAACAGGGTATCAGAATTCTC-3′ and reverse 5′-TGCTGACAATAGCCATTAGG-3′.

### 2.4 Cell proliferation assay

Twenty-four hours after being transfected, MG-63 and Saos-2 cells were seeded at 1 × 10^3^ cells per well in 96-well plates. The cell proliferation assay was performed on days 1–3 using the CellTiter 96 AQueous One Solution Cell Proliferation Assay kit (Promega). The proliferation rate was calculated using the following formula: Proliferation rate = (The absorbance of each time point ÷ The absorbance of 0 h − 1) × 100% (Same sample).

### 2.5 Transwell assay

The transwell assay was carried out as previously described [16]. Briefly, transfected cells (5 × 10^4^ cells) in 200 µL of 0.1% fetal bovine serum (FBS) containing medium were placed in the upper chamber. The lower chamber was filled with 10% FBS medium (600 µL). For invasion, Matrigel (BD Biosciences, San Diego, CA, USA) was precoated on the bottom of the upper chamber. Twenty-four hours later, cells on the upper chamber were removed, and the cells that passed through the membrane filter were fixed and stained. Finally, five photographs at 200× magnification were randomly collected using LEICA Microscope (Tokyo, Japan) for each well. The number of migrated and invaded cells in the five fields were counted, and the average was calculated.

### 2.6 Western blotting analysis

Western blotting was carried out as previously described [16]. Briefly, equal amounts of total proteins (about 30 µg) were separated using 10% SDS polyacrylamide gel electrophoresis and transferred onto a polyvinylidene difluoride membrane (Millipore, Billerica, MA, USA). After blocking, membranes were incubated with anti-activator of 90 kDa heat shock protein ATPase homolog 1 (AHSA1) antibody (at 1:1,000 dilution, Abcam, Cambridge, MA, USA) for 1 h at 37°C. After washing in TBS with 0.5% Tween 20 (TBST), the membrane was incubated with horseradish peroxidase-conjugated secondary antibody. Finally, chemiluminescent detection was performed using enhanced chemiluminescence (ECL), and signals were recorded on X-ray films.

### 2.7 Plasmid construction and luciferase reporter assay

The wild-type (WT) LINC00707 sequence was cloned into luciferase reporter vector psi-CHECK2 using the forward primer (5′-CCGCTCGAGAAGCAGGCAGAGGAACGTGGAAG-3′) and reverse primer (5′-ATAAGAATGCGGCCGCGGCTCAACAGTCAAAGACAGGTT-3′), and the recombinant plasmid was termed psiCHECK-LINC00707-WT. The binding site of miR-338-3p (ATGCTGG) on psiCHECK-LINC00707-WT was mutated into CGATGAA by a site-directed mutagenesis kit (Sangon Biotech, Shanghai, China), and the plasmid was termed psiCHECK-LINC00707-mut. For the luciferase reporter assay, 293 T cells were plated at 5 × 10^4^ cells per well in 24 well plates. Next day, psiCHECK-LINC00707-WT plus miR-338-3p mimics, psiCHECK-LINC00707-WT plus miR-NC, psiCHECK-LINC00707-mut plus miR-338-3p mimics, and psiCHECK-LINC00707-mut plus miR-NC were transfected into cells using Lipofectamine 2000 (Invitrogen). Forty-eight hours after transfection, luciferase assays were performed using the dual luciferase reporter assay system (Promega).

### 2.8 Statistical analysis

All assays were performed in triplicate. Statistical analysis was performed using SPSS 19.0 software package (IBM Corp, Armonk, NY, USA). Significance analysis was performed using Student *t*-tests. *P* < 0.05 was considered to indicate statistically significant differences.

## 3 Results

### 3.1 LINC00707 expression is increased in osteosarcoma cells

**Figure 1:**
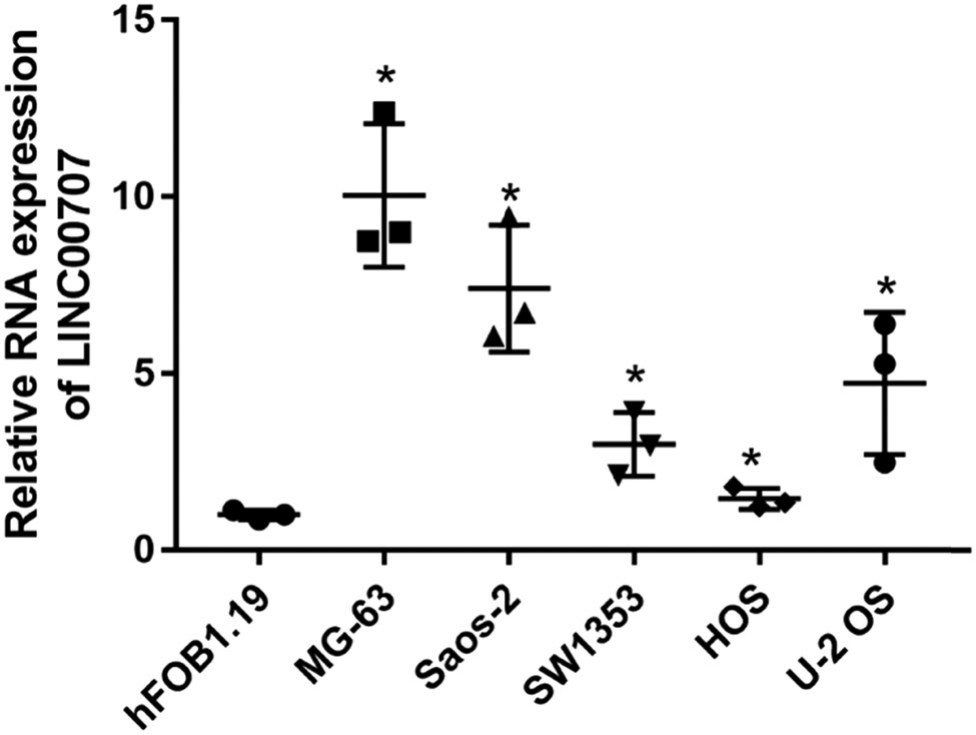
LINC00707 expression is increased in osteosarcoma cells. LINC00707 expression in the osteoblastic cell line hFOB1.19 and human osteosarcoma cell lines was detected by qRT-PCR. *P < 0.05.

To investigate whether LINC00707 is abnormally expressed in osteosarcoma, qRT-PCR was performed to determine LINC00707 levels in the osteoblastic cell line hFOB1.19 and human osteosarcoma cell lines. As shown in Figure 1, LINC00707 levels are significantly higher in osteosarcoma cell lines SW 1353, HOS, U-2 OS, MG-63, and Saos-2 compared to hFOB1.19. Moreover, the highest levels of LINC00707 were observed in MG-63 and Saos-2. Therefore, these two cell lines were selected for the following assays.

### 3.2 LINC00707 silencing suppress cell proliferation, migration, and invasion

**Figure 2:**
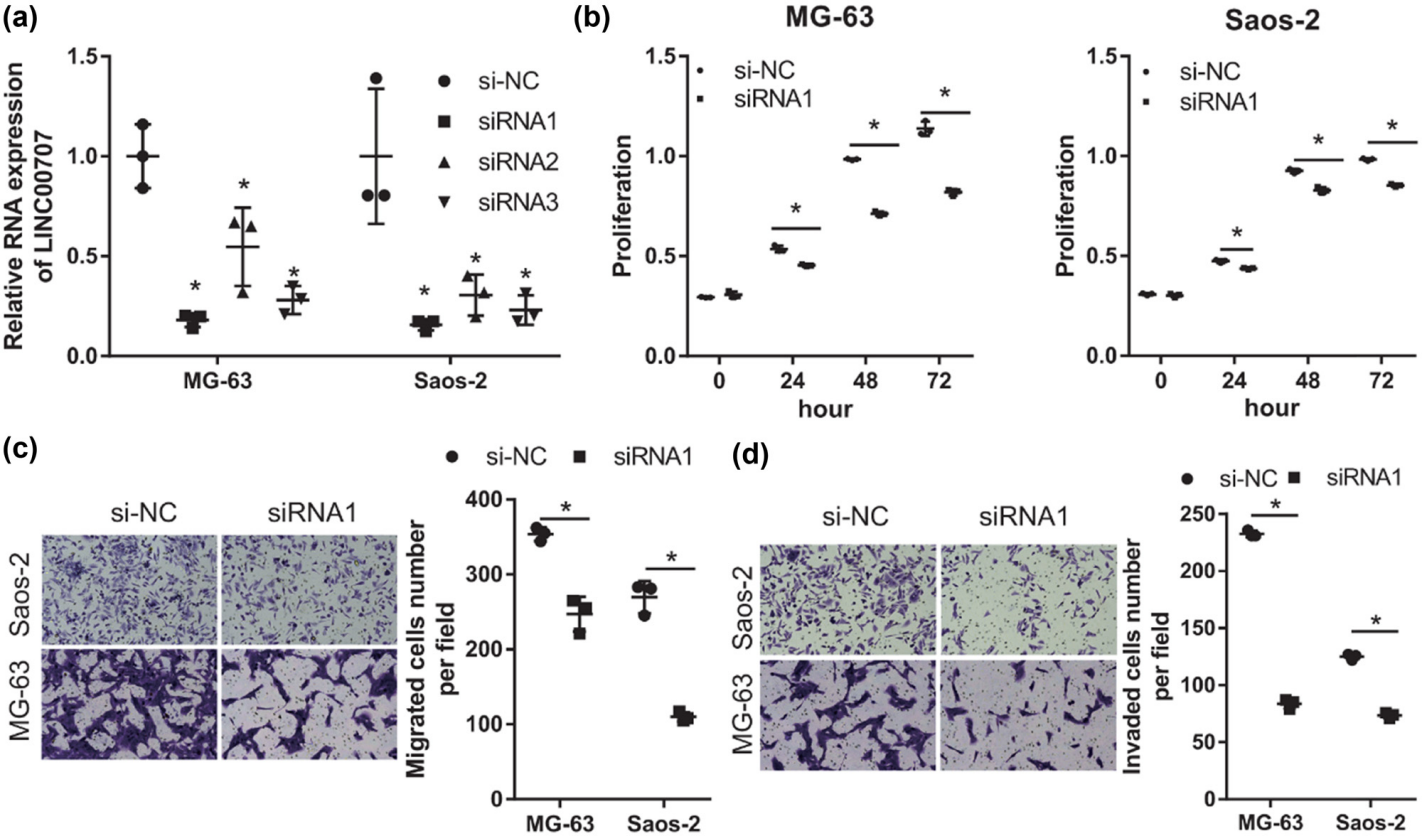
LINC00707 silencing suppressed cell proliferation, migration, and invasion. (a) LINC00707 levels in MG-63 and Saos-2 cells transfected with small interfering RNA (siRNA) targeting LINC00707 (siRNA1, siRNA2, and siRNA3) and negative control siRNA (si-NC) were measured by qRT-PCR after transfected at 24 h. (b) The effect of LINC00707 silencing by siRNA1 transfection on the proliferation of MG-63 and Saos-2 cells. (c and d) The effect of LINC00707 silencing by siRNA1 transfection on migration (c) and invasion (d) of MG-63 and Saos-2 cells. Migration as detected by the transwell assay and invasion was detected by the transwell-matrigel assay. (c and d) Left includes the photographs and right presents the quantification of the results of the migrated or invaded cell numbers per field. *P < 0.05.

To investigate the role of LINC00707 in osteosarcoma cells, three siRNA targeting LINC00707 (siRNA1, siRNA2, and siRNA3) were transfected into MG-63 and Saos-2 cells to knockdown LINC00707. As shown in Figure 2a, LINC00707 levels were significantly lower in MG-63 and Saos-2 cells transfected with siRNA1, siRNA2, or siRNA3 compared to cells transfected with si-NC. These results indicated that siRNAs targeting LINC00707 can successfully knockdown LINC00707. siRNA1, which had the highest interference efficiency, was selected for the subsequent experiments. Next, we investigated the effect of LINC00707 silencing on cell proliferation, migration, and invasion of MG-63 and Saos-2 cells. The results of the cell proliferation assay showed that the proliferation rate of the siRNA1 group of cells was lower than that of the si-NC group of cells (Figure 2b). The results of the transwell migration assay showed that the number of the migrated and invaded cells of the siRNA1 group was lower than that of the si-NC group (Figure 2c and d). These results revealed that LINC00707 silencing can suppress cell proliferation, migration, and invasion of MG-63 and Saos-2 cells.

In addition, the pcDNA3.1-LINC00707 (ov-LINC00707) and pcDNA3.1 (ov-NC) were transfected into MG-63 and Saos-2 cells to overexpress LINC00707. LINC00707 levels were significantly higher in MG-63 and Saos-2 cells in the ov-LINC00707 group compared to the ov-NC group (Figure S1a). Next, the cell proliferation and the migrated and invaded cells in the ov-LINC00707 group were significantly higher than that of the ov-NC group (Figure S1b–d). These results revealed that LINC00707 overexpression can promote cell proliferation, migration, and invasion of MG-63 and Saos-2 cells.

### 3.3 LINC00707 acts as a ceRNA of miR-338-3p

**Figure 3:**
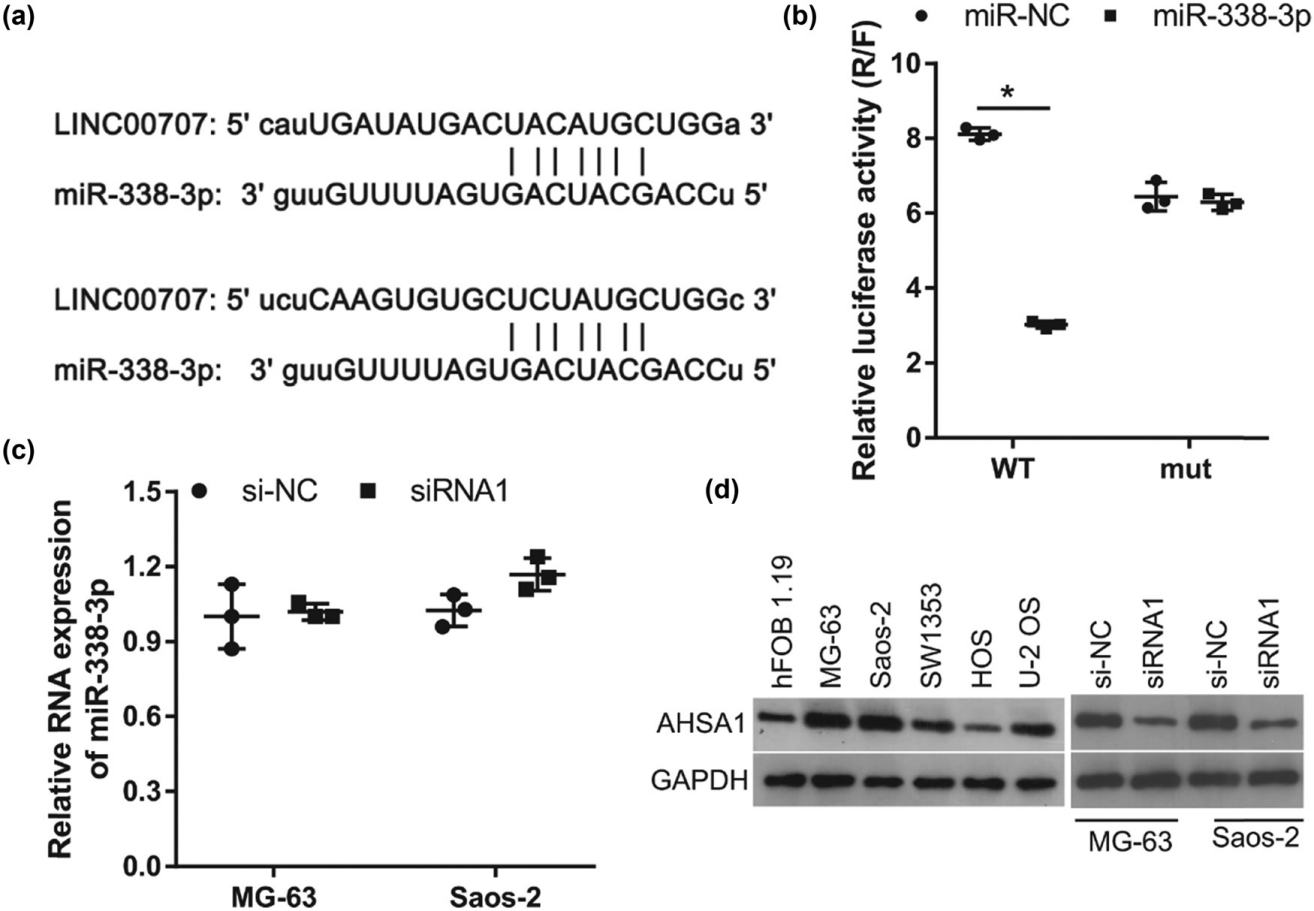
LINC00707 acts as a ceRNA of miR-338-3p. (a) A schematic diagram of miR-338-3p binding sites on LINC00707 sequence. (b) miR-338-3p cotransfection suppressed the relative luciferase activity of cells transfected with the luciferase reporter vector psi-CHECK2 containing wild-type (WT) LINC00707 sequence (psiCHECK-LINC00707-WT) compared to negative control miRNA (miR-NC). (c) miR-338-3p levels in MG-63 and Saos-2 cells transfected with small interfering RNA (siRNA) targeting LINC00707 (siRNA1) or negative control siRNA (si-NC). (d) AHSA1 protein levels in hFOB1.19, MG-63 and Saos-2 cells transfected with siRNA1 or si-NC. *P < 0.05.

To investigate the regulatory mechanism of LINC00707, we hypothesized that miRNAs may have binding sites on the LINC00707 sequence. As shown in Figure 3a, miR-338-3p, which inhibits proliferation, migration, invasion, and epithelial–mesenchymal transition (EMT) in osteosarcoma cells [16], has two binding sites on the LINC00707 sequence. The results of the luciferase reporter assay showed that co-transfection of miR-338-3p can decrease the relative luciferase activity of psiCHECK-LINC00707-WT transfected cells and had no effect on the relative luciferase activity of psiCHECK-LINC00707-mut transfected cells (Figure 3b), indicating that miR-338-3p can bind on LINC00707. Moreover, we found that miR-338-3p levels were not different between the si-NC and siRNA1 group of cells (Figure 3c). However, the expression of AHSA1, a target of miR-338-3p, was higher in MG-63 and Saos-2 cells than that in the hFOB1.19 cell (Figure 3d). AHSA1 expression was lower in the siRNA1 group of cells than that in the si-NC group of cells (Figure 3d). These results suggested that LINC00707 may act as a ceRNA of miR-338-3p to regulate AHSA1 expression.

### 3.4 miR-338-3p inhibitor alleviated the effect of LINC00707 silencing

**Figure 4:**
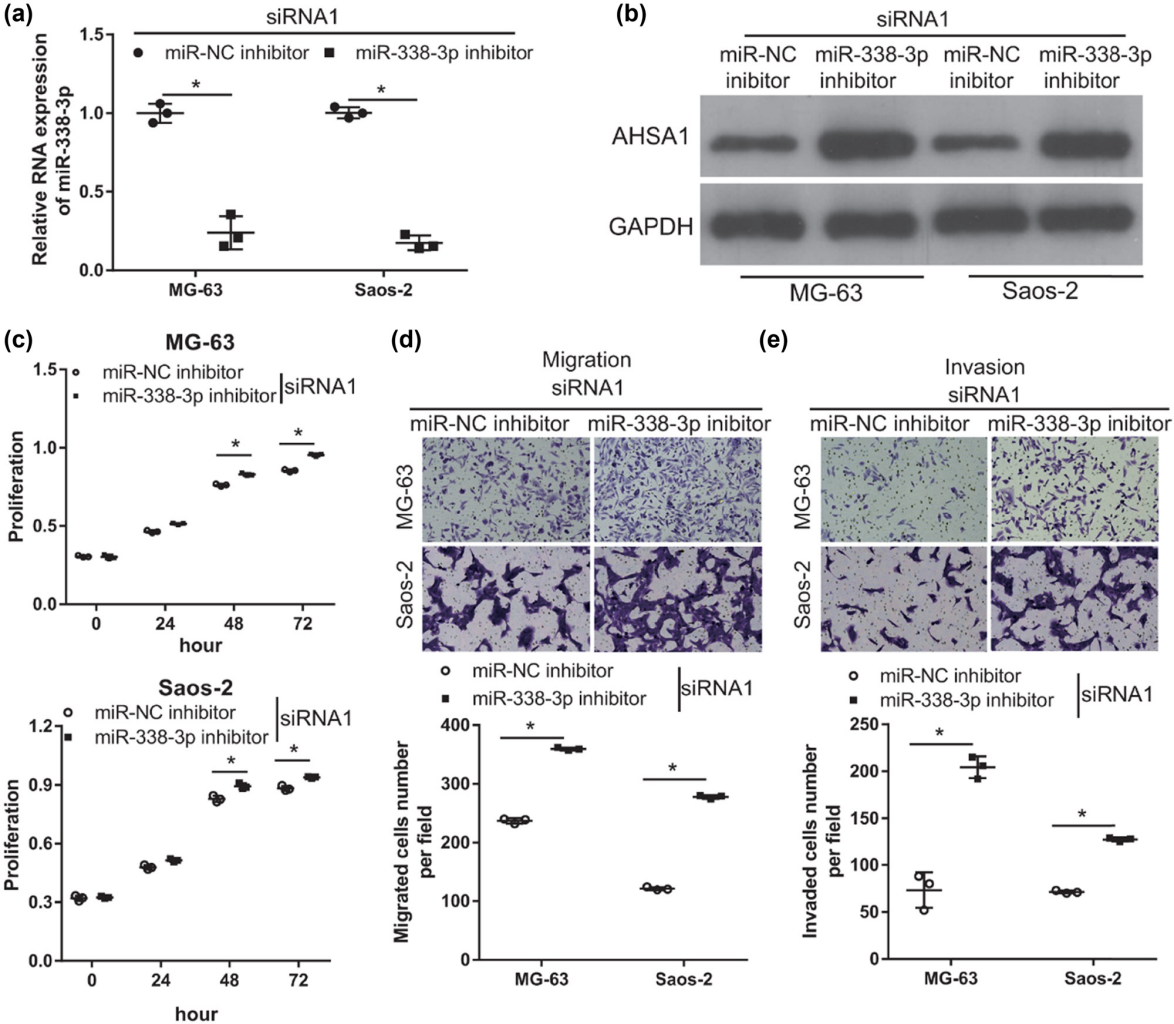
miR-338-3p inhibitor alleviated the effect of LINC00707 silencing. MG-63 and Saos-2 cells were cotransfected with miR-NC inhibitor plus siRNA1 or miR-338-3p inhibitor plus siRNA1. The effect of cotransfection on cell proliferation, migration, and invasion was evaluated. (a) miR-338-3p expression was measured by qRT-PCR after transfection at 24 h. (b) AHSA1 protein level was measured by western blot assay after transfection at 48 h. (c) Cell proliferation was examined by the MTS assay. (d and e) Migration was assayed by the transwell assay and invasion by the transwell-matrigel assay. The upper panel includes the photographs, and the lower panel shows the quantification and statistical analysis of the migrated or invaded cell numbers per field. *P < 0.05.

To verify whether LINC00707 plays its role by acting as a ceRNA of miR-338-3p, miR-338-3p inhibitor was co-transfected with siRNA1 (miR-338-3p inhibitor + siRNA1 group) to block the effect of LINC00707 silencing on miR-338-3p/AHSA1 axis. Cotransfection of miR-NC inhibitor and siRNA1 (miR-NC inhibitor + siRNA1 group) was performed as a control. miR-338-3p expression was obviously decreased in miR-338-3p inhibitor + siRNA1 group compared with that in miR-NC inhibitor + siRNA1 group (Figure 4a). AHSA1 expression was obviously increased in cells transfected with miR-338-3p inhibitor + siRNA1 compared with that in miR-NC inhibitor + siRNA1 (Figure 4b). In addition, the results of the cell proliferation assay showed that the proliferation rate of miR-338-3p inhibitor + siRNA1 group of cells was higher than that of miR-NC inhibitor + siRNA1 group of cells (Figure 4c). The results of the transwell assay showed that the number of migrated and invaded cells transfected with miR-338-3p inhibitor + siRNA1 was higher than that of cells transfected with miR-NC inhibitor + siRNA1 (Figure 4d and e). These results revealed that miR-338-3p inhibitor alleviated the effect of LINC00707 silencing on cell proliferation, migration and invasion, and AHSA1 expression of MG-63 and Saos-2 cells.

### 3.5 *AHSA1* overexpression alleviated the effect of LINC00707 silencing

**Figure 5:**
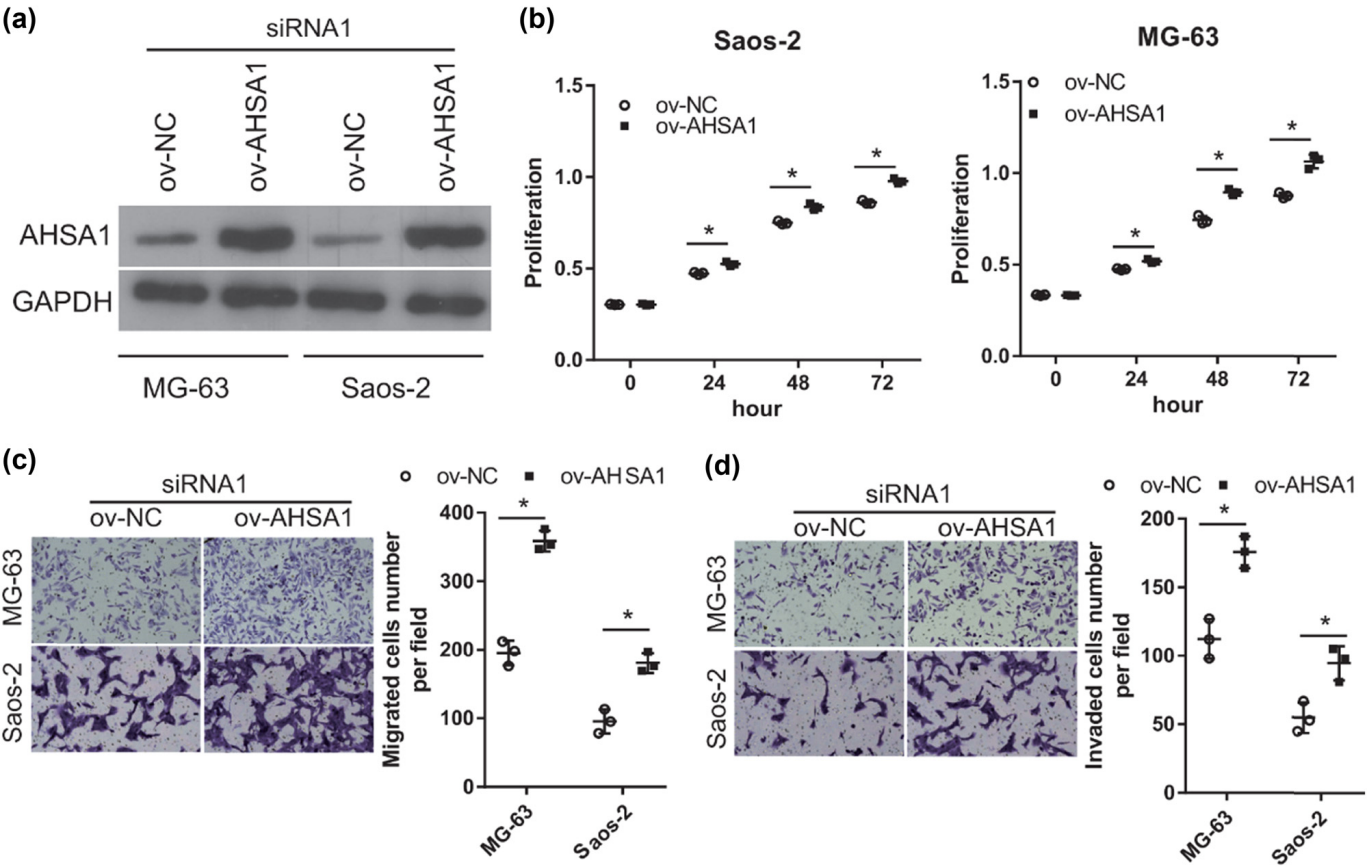
AHSA1 overexpression alleviated the effect of LINC00707 silenced. MG-63 and Saos-2 cells were cotransfected with ov-NC plus siRNA1 or ov-AHSA1 plus siRNA1. The effect of cotransfection on cell proliferation, migration, and invasion was evaluated. (a) AHSA1 expression was measured by western blot in MG-63 and Saos-2 cells cotransfected with miR-NC inhibitor plus siRNA1 or miR-338-3p inhibitor plus siRNA1. (b) AHSA1 expression was measured by western blot in MG-63 and Saos-2 cells cotransfected with ov-NC plus siRNA1 or ov-AHSA1 plus siRNA1. (c) Cell proliferation was examined by the MTS assay. (d and e) Migration was assayed by the transwell assay and invasion by the transwell-matrigel assay. The upper panel includes the photographs, and the lower shows the quantification and statistical analysis of the migrated or invaded cell numbers per field. *P < 0.05.

To verify the relationship between LINC00707 and AHSA1, AHSA1 expression was overexpressed in siRNA1 transfected MG-63 and Saos-2 cells (Figure 5b). The results of the cell proliferation assay showed that the proliferation rate of ov-AHSA1 + siRNA1 group of cells was higher than that of the ov-NC + siRNA1 group of cells (Figure 5c). The results of the transwell assay showed that the number of migrated and invaded cells transfected with ov-AHSA1 + siRNA1 was higher than that of cells transfected with ov-NC + siRNA1 (Figure 5d and e). These results revealed that AHSA1 overexpression alleviated the effect of LINC00707 silencing on cell proliferation, migration, and invasion of MG-63 and Saos-2 cells.

## 4 Discussion

Osteosarcoma is the most common type of primary malignant tumor of the bone, with a high metastasis rate and poor prognosis [1]. Therefore, it is important to further understand molecular mechanisms involved in the development of osteosarcoma and explore new molecular therapeutic targets. The abnormal expression of lncRNAs in human malignant tumors is being gradually recognized as a regulator of tumorigenesis and development [17,18]. Many lncRNAs, such as lncRNA OR3A4, lncRNA KCNQ1OT1, and LINC00514, have been demonstrated to be involved in osteosarcoma progression, drug resistance, and prognosis [19,20,21,22]. However, the roles of lncRNAs in osteosarcoma are still far from being fully understood. LINC00707 has been shown to be an oncogenic gene in several cancers [6,7,8]. However, its role and function in osteosarcoma are unclear. In the present study, we further clarified its role and regulatory mechanism in osteosarcoma. This study will provide the basis for gene-targeted therapy and may have important clinical significance.

We found that LINC00707 levels were higher in osteosarcoma cells than that in hFOB1.19 cells, suggesting that LINC00707 may be involved in osteosarcoma progression. Next, we examined the validity of this suggestion by silencing and overexpressing LINC00707 in osteosarcoma cell lines. Our results showed that LINC00707 knockdown suppressed cell proliferation, migration, and invasion of MG-63 and Saos-2 cells, whereas LINC00707 overexpression promoted cell proliferation, migration, and invasion of MG-63 and Saos-2 cells. These results indicated that LINC00707 is involved in osteosarcoma progression and is an oncogenic gene in osteosarcoma. Therefore, LINC00707 may be a therapeutic target for the osteosarcoma treatment. Our results are consistent with previous studies, which found that knockdown of LINC00707 significantly reduced cell proliferation and suppressed cell migration and invasion in lung adenocarcinoma and hepatocellular carcinoma [7,23]. All these studies suggested that abnormally expressed LINC00707 in human malignant tumors is a regulator of tumorigenesis and cancer development.

To elucidate the mechanism of LINC00707 in osteosarcoma, we examined whether it plays the function as a ceRNA. Among all the miRNAs that have binding sites on LINC00707 sequence, miR-338-3p attracted our attention. Our previous study has demonstrated that miR-338-3p can target-inhibit the expression of AHSA1 to inhibit proliferation, migration, invasion, and EMT in osteosarcoma cells [16]. Therefore, we predicted that LINC00707 is involved in osteosarcoma progression by sponging miR-338-3p. The results of the luciferase reporter assay indicated that LINC00707 can bind miR-338-3p. In addition, miR-338-3p inhibitor alleviated the effect of LINC00707 silencing in MG-63 and Saos-2 cells. These results suggest that silencing of LINC00707 suppresses cell proliferation, migration, and invasion by sponging miR-338-3p.

Next, we found that silencing LINC00707 can decrease the protein levels of AHSA1. miR-338-3p inhibitor enhanced AHSA1 expression in LINC00707-silenced MG-63 and Saos-2 cells. AHSA1 overexpression promoted cell growth, migration, and invasion in osteosarcoma cells by activating the Wnt/β-catenin signaling pathway [24]. In addition, AHSA1 overexpression alleviated the effect of LINC00707 silencing in MG-63 and Saos-2 cells. miR-338-3p inhibitor also enhanced AHSA1 expression in LINC00707-silenced MG-63 and Saos-2 cells. These results suggest that silencing of LINC00707 suppresses cell proliferation, migration, and invasion by inhibiting AHSA1 expression. Taken together, our results revealed that silencing LINC00707 suppresses cell proliferation, migration, and invasion by targeting the miR-338-3p/AHSA1 axis in MG-63 and Saos-2 cells.

Our present study has some limitations. First, we did not examine LINC00707 expression profiles in osteosarcoma tissues. Second, although we identified that LINC00707 functions by targeting miR-338-3p/AHSA1 axis, LINC00707 may have other potential targets. Further research is warranted to identify other potential targets of LINC00707. Third, we did not verify the role of LINC00707 in animals. Despite these drawbacks, our study highlights the potential role and the mechanism of LINC00707 in osteosarcoma.

In conclusion, we demonstrated that LINC00707 is highly expressed in osteosarcoma cell lines, and silencing of LINC00707 suppresses cell proliferation, migration, and invasion by targeting the miR-338-3p/AHSA1 axis in MG-63 and Saos-2 cells. These findings suggest that LINC00707 may serve as a potential target for osteosarcoma treatment.
